# Exogenous Putrescine Increases Heat Tolerance in Tomato Seedlings by Regulating Chlorophyll Metabolism and Enhancing Antioxidant Defense Efficiency

**DOI:** 10.3390/plants11081038

**Published:** 2022-04-11

**Authors:** Mohammad Shah Jahan, Md. Mahadi Hasan, Fahad S. Alotaibi, Nadiyah M. Alabdallah, Basmah M. Alharbi, Khaled M. A. Ramadan, Eslam S. A. Bendary, Dikhnah Alshehri, Dilfuza Jabborova, Doha A. Al-Balawi, Eldessoky S. Dessoky, Mohamed F. M. Ibrahim, Shirong Guo

**Affiliations:** 1 College of Horticulture, Nanjing Agricultural University, Nanjing 210095, China; shahjahansau@gmail.com; 2 Department of Horticulture, Faculty of Agriculture, Sher-e-Bangla Agricultural University, Dhaka 1207, Bangladesh; 3 State Key Laboratory of Grassland Agro-Ecosystems, College of Ecology, Lanzhou University, Lanzhou 730000, China; hasanmahadikau@gmail.com; 4 King Abdulaziz City for Science and Technology, Riyadh 12354, Saudi Arabia; falotaibi@kacst.edu.sa; 5 Department of Biology, College of Science, Imam Abdulrahman Bin Faisal University, Dammam 31441, Saudi Arabia; nmalabdallah@iau.edu.sa; 6 Department of Biology, Faculty of Science, University of Tabuk, Tabuk 71491, Saudi Arabia; b.alharbi@ut.edu.sa (B.M.A.); dalshehri@ut.edu.sa (D.A.); d.albalawi@ut.edu.sa (D.A.A.-B.); 7 Central Laboratories, Department of Chemistry, King Faisal University, Al-Ahsa 31982, Saudi Arabia; kramadan@kfu.edu.sa; 8 Department of Agricultural Biochemistry, Faculty of Agriculture, Ain Shams University, Cairo 11241, Egypt; esslam136@gmail.com; 9 Institute of Genetics and Plant Experimental Biology, Uzbekistan Academy of Sciences, Kibray 111208, Uzbekistan; dilfuzajabborova@yahoo.com; 10 Department of Biology, College of Science, Taif University, Taif 21944, Saudi Arabia; es.dessouky@tu.edu.sa; 11 Department of Agricultural Botany, Faculty of Agriculture, Ain Shams University, Cairo 11566, Egypt; ibrahim_mfm@agr.asu.edu.eg

**Keywords:** chlorophyll degradation, oxidative stress, photosynthesis, polyamines, thermotolerance, tomato

## Abstract

Crops around the world are facing a diversity of environmental problems, of which high temperatures are proving to be the most serious threat to crops. Polyamine putrescine (Put) acts as a master growth regulator that contributes to optimal plant growth and development and increased stress tolerance. Here, the current study aimed to elucidate how Put functions in regulating chlorophyll (Chl) metabolism, oxidative stress, and antioxidant defense, as well as to characterize the expression of genes related to heat stress in tomato seedlings under such stress. The results revealed that Put treatment significantly attenuates heat-induced damage by promoting biomass production, increasing photosynthetic efficiency, and inhibiting excessive production of oxidative stress markers. Heat stress markedly decreased the Chl content in the tomato leaf and accelerated the leaf yellowing process. However, Put-treated tomato seedlings showed a higher Chl content, which could be associated with the functions of Put in elevating PBGD activity (Chl biosynthesis enzyme) and suppressing the activity of the Chl catabolic enzyme (Chlase and MDCase). Under high-temperature stress, the expression levels of the gene encoding factors involved in Chl biosynthesis and Chl catabolism were significantly down- and upregulated, respectively, and this trend was reversed in Put-treated heat-stressed seedlings. In addition, exogenous application of Put boosted the activity of antioxidant enzymes, along with the levels of expression of their encoding genes, only in plants that were heat stressed. Furthermore, the expression levels of heat-shock-related genes (*HSP90*, *HSP70,* and *HsfA1*) were elevated in Put-treated, high-temperature-stressed tomato seedlings. Taken together, our results indicate that Put treatment significantly increases the heat tolerance of tomato seedlings, by elevating Chl concentrations and suppressing Chl catabolic enzyme activity, modulating endogenous free PA content, increasing antioxidant defense efficiency, and upregulating the expression of heat-shock-related genes.

## 1. Introduction

The tomato (*Solanum lycopersicum*) is one of the most important vegetable crops grown worldwide. Like most crops, tomato is sensitive to abiotic stresses. Heat stress is a major ecological constraint, which threatens world food security as global warming has progressed in recent times [1]. Global temperatures are predicted to increase by around 1.8~4.0 °C by 2100 [2]. An increase of one degree Celsius in annual temperature will result in a 2.5 to 16% reduction in crop yield [3]. Heat stress is regarded as a fatal abiotic stress because it affects crops at their morphological, physiological, biochemical, and cellular levels; limiting crop yield [4]. Numerous forms of physiological damage have been observed in plants exposed to high temperatures, including scorching of the plant leaves, suppression of shoot and root growth, leaf drop and senescence, and fruit decline, all of which result in reduced plant productivity [5]. Proline, glycine betaine, and soluble sugars are examples of primary metabolites that also accumulate in plants when exposed to thermal stress [6]. Plants exposed to thermal stress can produce reactive oxygen species (ROS) that cause oxidative damage and also alter the metabolism of carbohydrates, lipids, and proteins in cells [7]. Excess ROS production in plant tissues during stress results in damage to photosynthetic components, interruption of electron transfer from chloroplasts and mitochondria, and severe injury to biomembrane cells [8]. However, cellular elements are generally protected from ROS-caused damage via an adapted antioxidant defense system that nullifies the detrimental effects of oxidative stress [8]. Chlorophyll (Chl) is an important pigment that absorbs light energy and participates in the photosynthesis process in plants. There is a positive correlation between photosynthesis and Chl content in plant [9]. Chl content in plants has been reported to depend on an optimal balance between Chl biosynthesis and catabolism [10]. Heat stress disrupts the balance between Chl biosynthesis and catabolism in plants, reducing the Chl content in the leaves [10] and, finally, hindering plant growth, development, and productivity. Chl biosynthesis pathway regulation is a sophisticated process, influenced by diverse external environmental factors, such as temperature [11]. Furthermore, internal transcriptional regulation is critical for Chl biosynthesis. Correspondingly, Chl catabolism is a significant physiological process in plants [12]. Numerous abiotic factors can aid in Chl catabolism in plants, including high temperature [13].

The devastating effects of thermal stress on Chl and the photosynthetic machinery is related to excess ROS production [14]. Excess temperature decreases photosynthetic and respiratory activity, due to an increase in chlorophyllase activity and a decrease in the amount of photosynthetic pigments [15]. Heat stress inhibits the functions of enzymes of carbon metabolism, sucrose synthesis, and carbohydrate metabolism, by suppressing the expression of genes related to carbohydrate metabolism [16]. Heat stress causes the production of heat shock proteins (HSPs), which act as molecular chaperones to prevent protein degradation [17]. Similarly, diverse heat shock proteins (HSP70 and HSP90) binding with molecular chaperones allow HSFs to stimulate heat stress responses [17]. Ohama et al. [18] demonstrated that the central region of HsfA1d, along with several other *Arabidopsis* HsfA1s, is a significant regulatory domain that inhibits the HsfA1d transactivation activity of HsfA1d by engaging with heat shock protein 70 (HSP70) and HSP90.

Polyamines (PAs) are composed of low-molecular-weight aliphatic nitrogenous bases, and their metabolic functions are primarily involved in response to stresses [19]. These responses might be due to the ability of PAs to adjust osmosis and detoxify the cell by scavenging ROS through enhancing antioxidant defense capacity or inhibiting ROS generation [20]. Pretreatment with Spm enhances tolerance to concurrent drought and HT stresses by stimulating the activity of antioxidant enzymes and quenching ROS detoxification, upregulating the expression of stress-related genes that prevent trifoliate orange seedlings from damage [21]. Additionally, polyamines may serve as signaling molecules to promote gene expression and enhance the DNA binding activity of transcription factors in stress environments [22]. Indeed, several studies have found that when plants are subjected to heat stress, they produce higher levels of free and bound polyamines [23,24]. Furthermore, recent research showed that polyamines can be used to improve thermal stress tolerance, enabling a higher yield production and better quality of final plant products [25,26]. Polyamines are, in nature, generally found as free molecules, but they are also found as conjugated or bound forms. Previous research has demonstrated that exogenous PAs can rapidly enter intact chloroplasts to assist in protecting the photosynthetic apparatus under adverse conditions [27]. Exogenous spraying of PAs was observed to considerably improve the photosynthetic attributes and the Chl fluorescence features of wheat under heat stress [28]. Putrescine is the central compound in the PA biosynthesis pathway, comprising two amino groups and acting as a synthetic precursor of spermidine and spermine. Putrescine can induce alterations in the plasma membrane of guard cells through controlling the size of potassium channel pores, to regulate pore opening and closing, restricting evaporation in the plant [29,30]. Exogenous application of Put can stimulate physiological activities and activate osmotic adjustment compounds in plants such as proline, total soluble sugars, and amino acids [30]. Recently, it was shown that the catabolic activity of Put could compensate for the loss of total chlorophyll and chlorophyll fluorescence in salt-stressed ginseng plants, implying that it protects seedlings from stress-related damage and restores their morphophysiological processes [31]. The cucumber leaves of plants treated with Put had elevated photochemical efficiency via an improved heat dissipation machinery, which prevented irreversible photoinhibition [32]. Moreover, Put is well known for its ability to alleviate salt stress in plants by boosting photosynthetic efficiency [33]. Put was shown to be inextricably linked with salinity stress and reduced overaccumulation of starch in cucumber plants [34]. Foliar spraying of Put mitigates the negative effect of high temperature on cotton flowers and fruit development by enhancing the amount of Put content in cotton flowers, which was associated with a higher seed set [35]. The putative functions of the exogenous addition of Put in mitigating stress damage have been well documented in some plants [30,36,37,38]. However, the underlying mechanisms by which Put controls Chl biosynthesis and mitigates heat stress in the model tomato plant remain largely unknown. The present investigation was, therefore, carried out to address this lack of knowledge. Here, we found that the application of Put enhanced Chl metabolism and antioxidant defense ability, as well as elevating the expression of stress-related genes, resulting in an improved heat tolerance of tomato seedlings.

## 2. Results

### 2.1. Effects of Putrescine on the Growth Attributes of Tomato Seedlings under Heat Stress

Exposure of plants to heat stress significantly limited plant growth and biomass production (Figure S1). As shown in Figure S1, plants under heat stress alone showed significant decreases in plant height (46%), stem diameter (34%), fresh shoot weight (33%), fresh root weight (23%), dry shoot weight (42%), and dry root weight (30%) compared to control seedlings. On the contrary, the application of Put significantly reduced the heat-induced growth retardation and increased all the growth and biomass attributes of tomato seedlings under thermal stress (Figure S1). 

### 2.2. Putrescine Treatment Increases Pigment Content and Regulates Chlorophyll Metabolism in Tomato Seedlings under Heat Stress 

**Figure 1:**
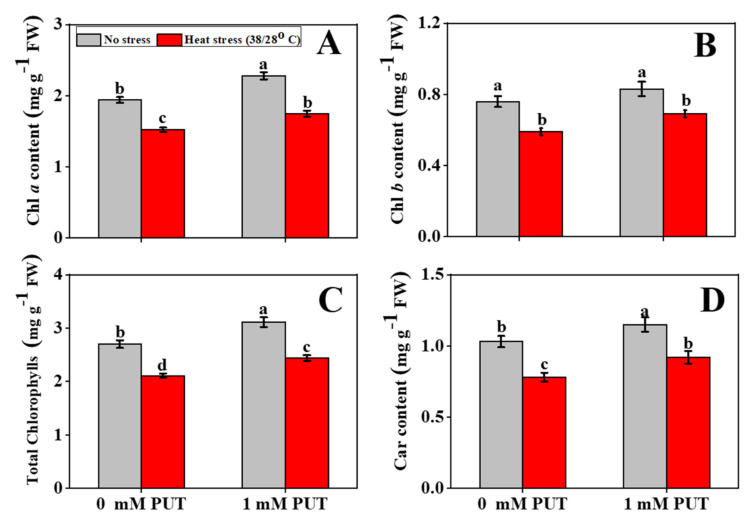
Interactive effects of Putrescine (Put) and high temperature on Chlorophyll (Chl) and carotenoid (Car) content in tomato seedlings. (**A**) Chl *a* content, (**B**) Chl *b* content, (**C**) Total Chl content, and (**D**) Car content. The data denote the mean value ± standard error (n = 3). Different alphabetic letters represent the significant differences among the treatments at *p* < 0.05, according to Tukey’s test.

High-temperature stress results in a sharp declined in pigment and carotenoid contents in tomato seedlings. The levels of Chl *a*, Chl *b*, total Chl, and carotenoids decreased by 22%, 22%, 21.8%, and 24%, respectively, only in the heat-stressed seedlings, in contrast to the corresponding controls (Figure 1A–D). By contrast, application of exogenous Put with heat-stressed seedlings restored Chl *a*, Chl *b*, total Chl, and carotenoid contents, in contrast to heat stress alone, which were 15%, 17%, 16%, and 18% more, respectively, indicating that the Put treatments significantly ameliorated heat stress-induced Chl degradation.

**Figure 2:**
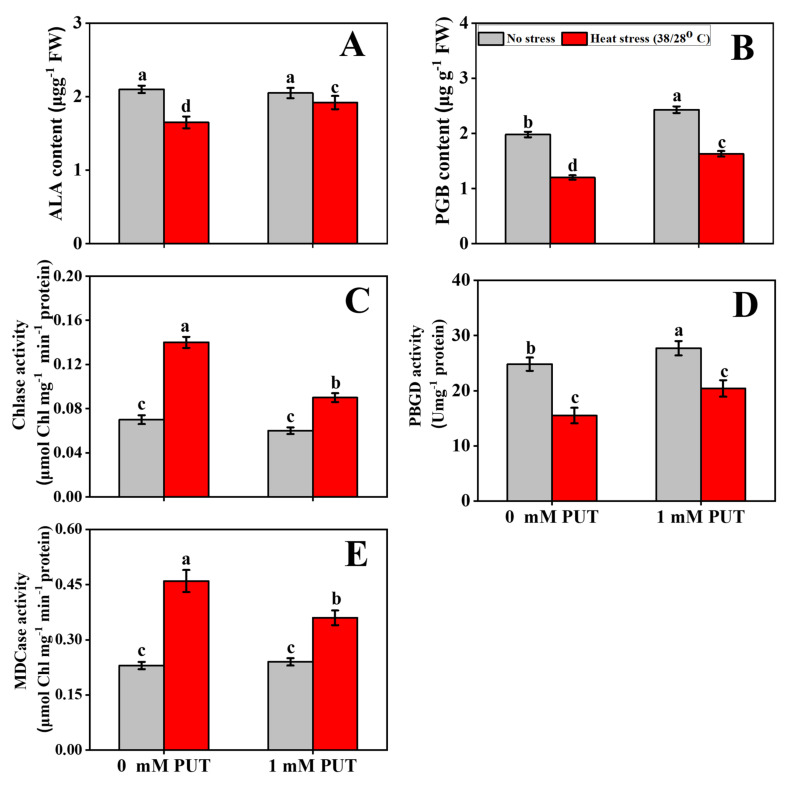
Interactive effects of Putrescine and high temperature on (**A**) δ-Aminolevulinic acid (ALA) content, (**B**) Porphobilinogen (PBG) content, (**C**) Chlorophyllase (Chlase) activity, (**D**) Porphobilinogen deaminase (PBGD) activity, and (**E**) Mg-dechelatase (MDCase) activity in tomato seedlings. The data denote the mean value ± standard error (n = 3). Different alphabetic letters represent the significant differences among the treatments at *p* < 0.05, according to Tukey’s test.

**Figure 3:**
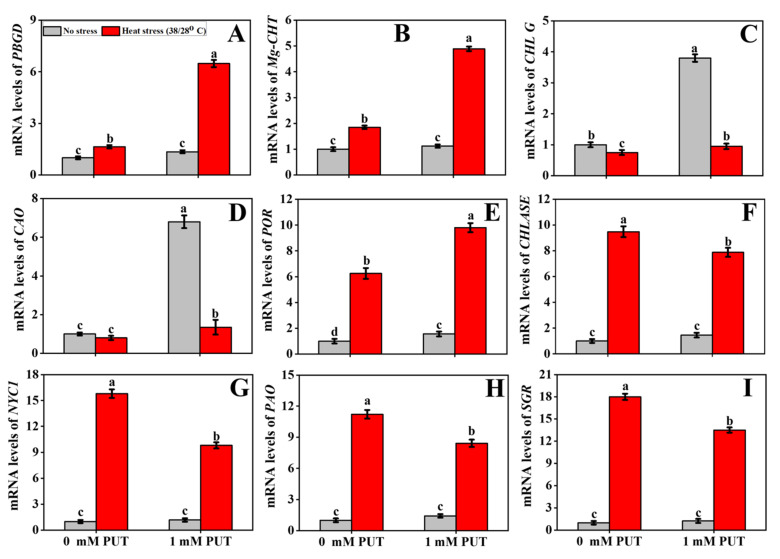
Interactive effects of Putrescine and high temperature on Chlorophyll related genes expression in tomato seedlings. (**A**) Porphobilinogen deaminase (*PBGD*), (**B**) Mg-chelatase (*Mg-CHT*), (**C**) Chl synthase (*CHL G*), (**D**) Chlorophyllide a oxygenase (*CAO*) activity, (**E**) protochlorophyllide oxidoreductase (*POR*), (**F**) Chlorophyllase (*CHLASE*), (**G**) nonyellow coloring (*NYC1*), (**H**) pheophorbide a oxidase (*PAO*), and (**I**) stay-green (*SGR*). The data denote the mean value ± standard error (n = 3). Different alphabetic letters represent the significant differences among the treatments at *p* < 0.05, according to Tukey’s test.

At controlled temperature, the application of exogenous Put on leaves significantly elevated the PBGD activity; however, heat stress markedly inhibited the activity of PGBD (Figure 2B). Heat stress significantly induced the inhibition of PBGD activity, which increased after Put treatment of heat-stressed tomato seedlings, in addition to upregulating the levels of porphobilinogen deaminase (*PBGD*) transcription in tomato leaves (Figure 3). These findings further denote that heat stress stimulates Chl biosynthesis and, thus, there was reduced contents of the chlorophyll precursors PBG and ALA (Figure 2A,B). Alternatively, the ALA and PBG contents significantly increased in Put-treated tomato seedlings. To verify the genetic makeup, we also characterized other vital Chl biosynthesis-related enzyme genes, such as *CHL G* (encoding Chl synthase), *CAO* (chlorophyllide a oxygenase), *POR* (encoding protochlorophyllide oxidoreductase), and Mg-chelatase (*Mg-CHT*) (Figure 3). The reduction in Chl biosynthesis in plants subjected to elevated temperatures is accompanied by a decrease in the activity of enzymes involved in Chl biosynthesis [39]. The expression of biosynthesis-related genes was significantly increased in Put-treated seedlings, while the transcript abundances of these genes were significantly downregulated, only in heat-stressed seedlings (Figure 3). 

The two most important chlorophyll catabolism enzymes are Chlase and MDCase. No significant differences were observed in the their activities between the control and Put-treated seedlings grown at ambient temperature (Figure 2C,E). However, heat stress significantly increased Chlase activity and upregulated the transcript abundance of its encoding gene (chlorophyllase, *CHLASE*), compared with that of the control seedlings (Figure 3). These findings demonstrate that the Chl degradation rate was significantly elevated under thermal stress conditions. Foliar application with Put significantly increased the Chlase and MDCase activities, and their encoding gene expression was markedly reduced in tomato seedlings exposed to heat treatment (Figure 2C,E). We further quantified the expression of other signatory chlorophyll catabolic genes, namely nonyellow coloring (*NYC1*), senescence-inducible chloroplast stay-green (*SGR*), and pheophorbide a oxidase (*PAO*), which were significantly upregulated under heat stress, whereas Put with heat-treated seedlings significantly decreased the expression of the above mentioned genes (Figure 3), indicating that supplementation with Put slows down Chl degradation.

### 2.3. Effects of Putrescine on Gas Exchange Parameters under Heat Stress

**Figure 4:**
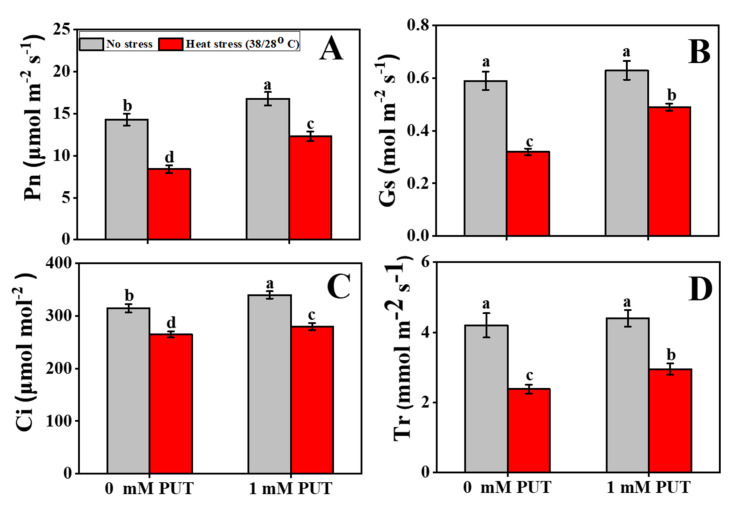
Interactive effects of Putrescine and high temperature on gas exchange parameter content in tomato seedlings. (**A**) Net photosynthetic rate (Pn) content, (**B**) Stomatal conductance (Gs) content, (**C**) Intercellular carbon dioxide (CO_2_) concentration (Ci) content, and (**D**) Transpiration rate (Tr) content. The data denote the mean value ± standard error (n = 3). Different alphabetic letters represent the significant differences among the treatments at *p* < 0.05, according to Tukey’s test.

High-temperature-stressed significantly reduced the gas exchange parameters values such as Pn, Gs, Ci, and Tr (Figure 4A–D). The values of Pn, Gs, Ci, and Tr decreased by 41%, 46%, 16%, and 43%, respectively, in plants under heat stress, compared to the corresponding control plants. However, combined treatment of plants with both Put and heat stress reduced the adverse effects on photosynthetic properties; and compared with only heat-stressed seedlings, the application of Put to heat-stressed seedlings limited the deleterious effects of Pn, Gs, Ci, and Tr to 46%, 53%, 7%, and 24%, respectively (Figure 4A–D).

### 2.4. The Positive Role of Putrescine on Photosynthesis-Related Attributes under Heat Stress

**Figure 5:**
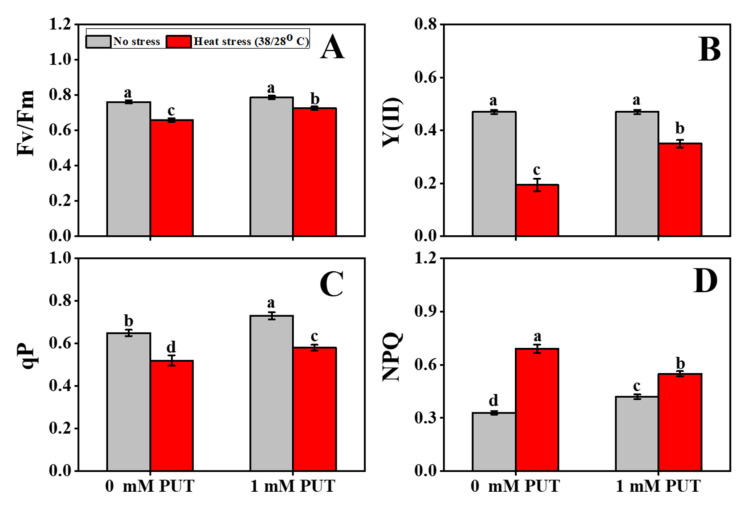
Interactive effects of Putrescine and high temperature on photosynthetic attributes in tomato seedlings. (**A**) Maximum quantum yield of PSII (Fv/Fm), (**B**) Effective quantum efficiency of PS II (Y(II)), (**C**) Photochemical quenching coefficient (qP), and (**D**) Non-photochemical quenching (NPQ). The data denote the mean value ± standard error (n = 3). Different alphabetic letters represent the significant differences among the treatments at *p* < 0.05, according to Tukey’s test.

To assess the impact of different light systems on thermal stress with Put, the maximum quantum yield of photosystem II (Fv/Fm) was determined. Prior to heat stress, the Fv/Fm value was no more affected by Put treatment than in the control plants (Figure 5). Under normal environments, the Fv/Fm ratio was 0.76, but this value was significantly reduced under heat stress (to 0.62). In addition, the value was increased again in plants treated with Put exposed to heat stress, and was 0.72. The effective quantum efficiencies of the PSII (Y(II)), NPQ (nonphotochemical quenching), and qP (photochemical quenching coefficient) of tomato leaves were also evaluated, to describe how photosystem II functions under heat stress and how Put affects the growth system of tomato plants (Figure 5). The results revealed that following Put and heat treatment, the effective photosynthetic quantum yield of nontreated plants decreased to 59%, while under heat stress with Put, the value of Y(II) increased by 1.82-fold compared with the control. Compared to the control treatment, the value of NPQ of the heat treatment without Put was 1.88-fold higher, but significantly lower in heat-stressed seedlings that had been sprayed with exogenous Put. In contrast to NPQ, qP was impaired at the level of the photosynthetic apparatus; the results showed a slight difference among the four treatments (Figure 5).

### 2.5. Effect of Putrescine on the Content of Proline and Soluble Sugar in Tomato Seedlings under Heat Stress

**Figure 6:**
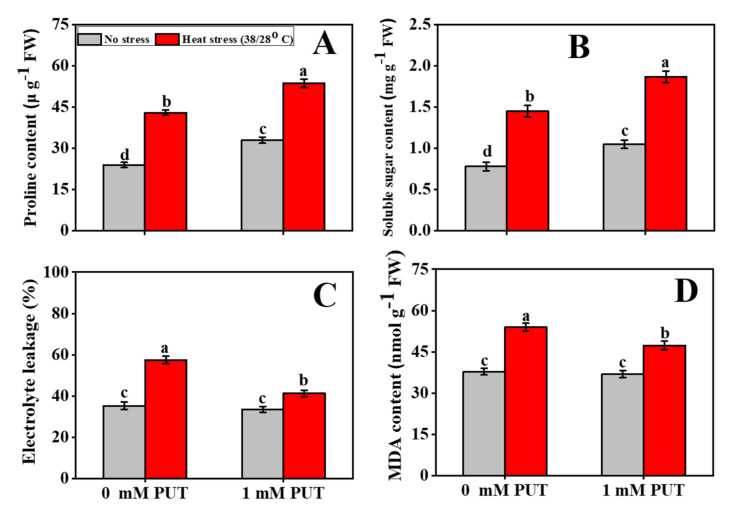
Interactive effects of Putrescine and high temperature on (**A**) Proline content, (**B**) Soluble sugar content, (**C**) Electrolyte leakage, and (**D**) MDA content in tomato seedlings. The data denote the mean value ± standard error (n = 3). Different alphabetic letters represent the significant differences among the treatments at *p* < 0.05, according to Tukey’s test.

The proline content increased significantly with high-temperature treatment, and it was 79% higher than the control treatment (Figure 6A). In comparison to high-temperature treatment, the application of Put significantly increased the content of proline. Treatment with 1 mM Put was the most efficient at increasing soluble sugar content compared to the other treatments, with an increase of 29%. The soluble sugar level was significantly reduced only in heat-stressed seedlings (Figure 6B).

### 2.6. Putrescine Treatment Mitigates the Oxidative Stress of Tomato Seedlings under Heat Stress

Relative electrolyte leakage is a marker of cellular membrane injury, and it was also significantly lower in Put-treated leaves than in controls (Figure 6C). The highest REL was observed in heat-stressed only seedlings. The MDA content in heat-stressed tomato seedlings was higher than that of other treatments and was significantly reduced in Put-treated tomato seedling under thermal stress conditions (Figure 6D). This suggests that Put treatment can reduce cell membrane damage and lipid peroxidation in tomato plants under heat stress. 

**Figure 7:**
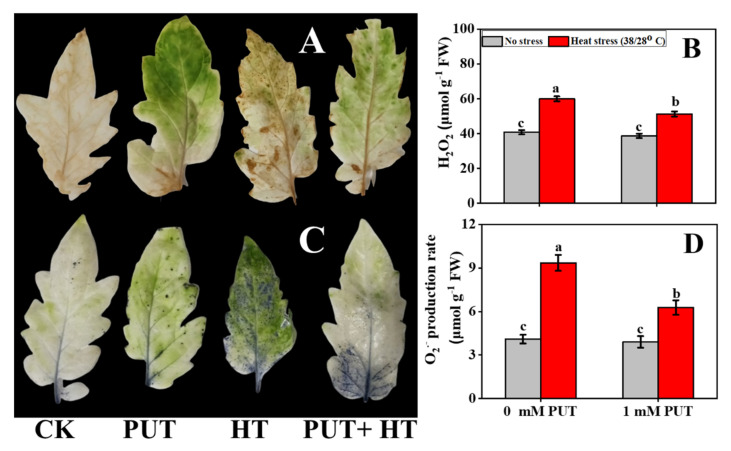
Interactive effects of Putrescine and high temperature on (**A**) Accumulation of hydrogen peroxide, (**B**) Hydrogen peroxide content, (**C**) Accumulation of superoxide anion, and (**D**) Superoxide anion production rate in tomato seedlings. The data denote the mean value ± standard error (n = 3). Different alphabetic letters represent significant differences among the treatments at *p* < 0.05, according to Tukey’s test.

To obtain further relevant data for establishing a theoretical basis for the observations, the underlying mechanisms related to the stress tolerance conferred by Put were further investigated (Figure 7). As stated previously, excessive accumulation of ROS is a typical response to environmental stresses. To ascertain whether Put treatment mitigates ROS production, the levels of two major ROS representatives, H_2_O_2_ and O_2_^•−^, were detected. Here, the concentrations of H_2_O_2_ and O_2_^•−^ in the leaves are denoted according to depth area as the number of brown and blue spots, respectively (Figure 7A,C). 

The brown spots became more evident in heat-treated seedlings. However, this symptom was not apparent in the leaves treated with Put, even after stress treatment. NBT staining also yielded similar results. Significantly fewer spots were observed in leaves treated with Put than in control leaves (Figure 7A,C). These results indicate that the seedlings treated with Put accumulated significantly less H_2_O_2_ and O_2_^•−^ than plants under only heat stress conditions. This is inextricably linked to the lower REL found in these samples. The concentration of H_2_O_2_ and O_2_^•−^ showed a similar trend as detected by histochemical staining, where higher levels of H_2_O_2_ and O_2_^•−^ were observed only in heat-stressed tomato plants, and not the other treatments (Figure 7B,D). The concentration of these stress markers was markedly reduced in Put-treated heat stressed seedlings, indicating that Put can mitigate oxidative stress by reducing the accumulation of stress marker components under thermal conditions.

### 2.7. Putrescine Increases Antioxidant Enzymes Activity under Heat Stress

**Figure 8:**
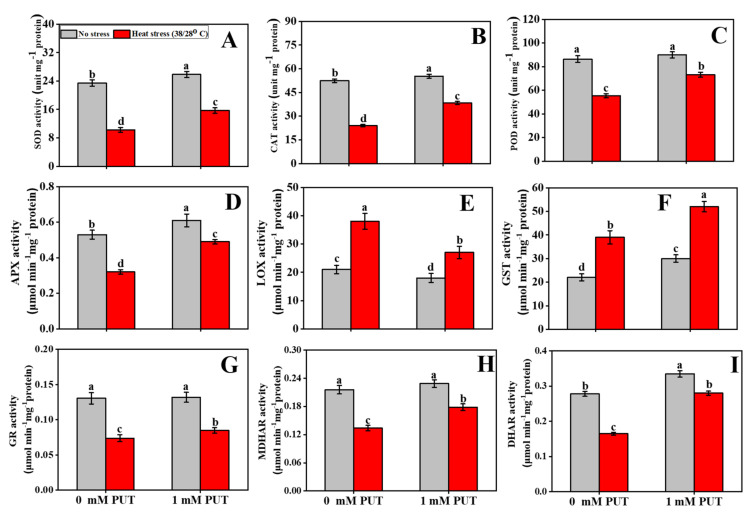
Interactive effects of Putrescine and high temperature on antioxidant enzymes activity in tomato seedlings. (**A**) Superoxide dismutase (SOD) activity, (**B**) Catalase (CAT) activity, (**C**) Peroxidase (POD) activity, (**D**) Ascorbate peroxidase (APX) activity, (**E**) Lipoxygenase (LOX) activity, (**F**) Glutathione S-transferase (GST) activity, (**G**) Glutathione reductase (GR) activity, (**H**) Monodehydroascorbate reductase (MDHAR) activity, and (**I**) Dehydroascorbate reductase (DHAR) activity. The data denote the mean value ± standard error (n = 3). Different alphabetic letters represent significant differences among the treatments at *p* < 0.05, according to Tukey’s test.

To evaluate whether Put is implicated in the attenuation of ROS-induced oxidative stress under heat stress, we examined the activity of antioxidant enzymes following treatment with or without Put and exposure of plants to thermal stress. The foliar application of Put had a beneficial role on enzyme activities in plants subjected to heat stress (Figure 8). The activity of SOD under heat stress was significantly decreased among all other treatments, but the activity increased greatly in seedlings subjected to combined heat stress and Put treatment (Figure 8A). Heat-stressed seedlings had reduced antioxidant enzyme activities of POD, CAT, and APX compared to control plants (Figure 8B–D), which decreased by 36%, 54%, and 65%, respectively, compared with control seedlings. The activities of POD, CAT, and APX were higher among plants sprayed with Put in the absence of heat stress than in normal plants (Figure 8B–D). Concurrently, the maximum activities of POD, CAT, and APX increased by 32%, 38%, and 35%, respectively, under the same level of heat stress with Put, compared to the heat stress only conditions. A significant increase, of 80%, in LOX activity was obtained with heat treatment compared to the control. However, Put treatment reduced the LOX activity in heat-stressed plants (Figure 8E). Temperature stress increased glutathione S-transferase (GST) activity by 77% in tomato seedlings. Put treatment together with heat stress resulted in an additional increase in this activity (Figure 8F).

No statistically significant differences were observed in GR activity between control and Put-treated seedlings (Figure 8G). However, the GR activity was reduced by 45% in heat-stressed plants compared with control seedlings. On the contrary, plants treated with Put and subsequently exposed to high temperatures showed an upregulation of GR activity by 30% compared to seedlings exposed to heat stress without Put pretreatment. Heat-stressed seedlings, on the other hand, showed a significant decrease in MDHAR and DHAR activities compared with other treated seedlings. Moreover, MDHAR and DHAR enzyme levels increased by 33% and 70%, respectively, in Put-treated heat-stressed seedlings compared with untreated heat-stressed seedlings (Figure 8H,I). 

### 2.8. Putrescine Modulates the Transcription of Antioxidant-Related Genes under Heat Stress

**Figure 9:**
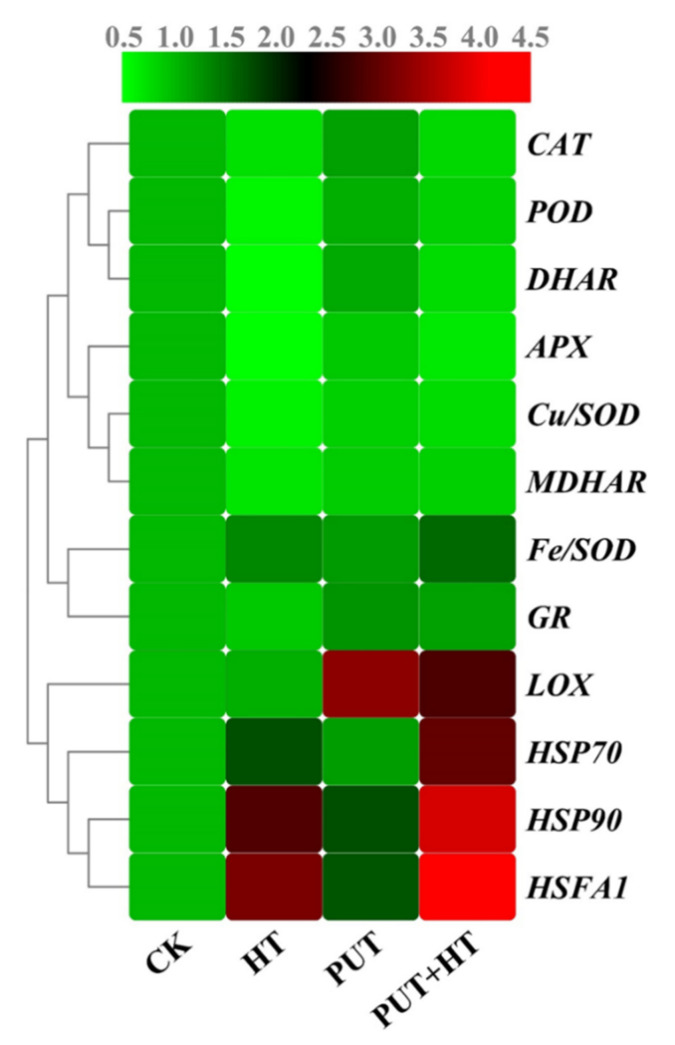
Heat map showing the expression of different stress-related genes in tomato leaves exposed to high temperature in the presence or absence of putrescine treatment. The intensity of gene expression ranges from deep green (low) color to deep red color (high). CK: control; Put: 1 mM putrescine; HT: heat stress (38/28 °C); Put + HT: 1 mM putrescine and heat stress. Antioxidant-related genes (*FeSOD, MnSOD, CAT, POD, APX, GR, MDHAR, DHAR, GST, LOX*); and Heat shock-related genes (*HSP90, HSP70, HSfA1*).

To illustrate the molecular framework by which Put mitigates heat stress-induced oxidative damage, the expression patterns of several key genes that encode antioxidant enzymes were investigated. Expression levels of most antioxidant genes were significantly decreased under heat stress. The additional supplementation of Put resulted in a notable increase in the transcript abundance of these genes under high temperature. Interestingly, the expression patterns of almost all of these genes were higher in the Put-treated seedlings. The results showed that the expression of the *Cu/SOD, POD*, *CAT*, *APX*, *MDHAR*, *DHAR,* and *GR* genes in heat-stressed seedlings was decreased by 48%, 52%, 35%, 57%, 38%, 57%, and 15%, respectively, compared to the corresponding control groups (Figure 9). Alternatively, the application of exogenous Put increased the expression of *Cu/SOD*, *POD*, *CAT*, *APX*, *MDHAR DHAR*, and *GR* by 1.33, 1.65, 1.11, 1.37, 1.27, 1.64, and 1.47 times, respectively, compared with untreated heat-stressed seedlings. Interestingly, the expression of *Fe/SOD* was reversed compared to the expression of other antioxidant genes. *LOX* expression was upregulated under heat stress conditions; however, this upregulation was significantly suppressed by Put application. These results indicate that exogenous Put can mitigate heat stress-induced oxidative damage by altering antioxidant defenses in heat-stressed tomato seedlings (Figure 9).

### 2.9. Putrescine Modulates Transcript Levels of Heat-Shock-Related Genes 

Heat shock proteins (HSPs) play an important role in ROS uptake, and heat shock transcription factors regulate the expression of heat shock proteins when exposed to high temperatures. Compared to control seedlings, the transcript abundances of *HSP70*, *HSP90,* and *HsfA1* were upregulated by 57%, 84%, and 87%, respectively, in seedlings subjected to only heat stress (Figure 9). Under normal conditions, supplementation with Put had no effect on the transcriptional levels of these genes. Conversely, Put treatment significantly upregulated the transcript levels of *HSP70*, *HSP90*, and *HsfA1* in plants exposed to thermal stress, and their expression was higher by 2.68-, 2.03-, and 2.01-fold, respectively, than heat-stressed only seedlings. Among these genes, the expression level of *HsfA1* in plants treated with Put was 15.87 times higher than that of control plants (Figure 9). 2.10. Effects of Putrescine on Endogenous Polyamine Levels under Heat Stress

**Figure 10:**
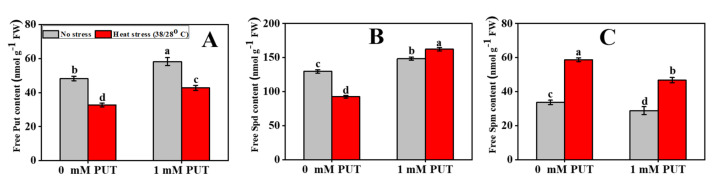
Interactive effects of Putrescine and high temperature on free polyamine content in tomato seedlings. (**A**) Free putrescine (Put) content, (**B**) Free spermidine (Spd) content, and (**C**) Free spermine (Spm) content. The data denote the mean value ± standard error (n = 3). Different alphabetic letters represent the significant differences among the treatments at *p* < 0.05, according to Tukey’s test.

Under unstressed conditions, exogenous Put significantly increased the content of free Put, by 24.4% and Spd by 00%, while it decreased the level of free Spm by 9.5% (Figure 10). However, the content of free Put and Spd decreased by 00% in the heat-stressed only seedlings compared to in the control seedlings, and the content of Spm increased by 00%. Moreover, under high-temperature stress, exogenous Put significantly increased the contents of free Put and Spd, to 1.3- and 1.75-fold, respectively, but decreased the Spm content by 20% compared to the heat-stressed only plants (Figure 10).

## 3. Discussion

The photosynthetic organ is one of the parts of a plant most vulnerable to thermal stress [40]. Parallel to other stresses, heat stress also causes water scarcity, induces oxidative stress, and reduces Chl concentrations in plants, in addition to restricting plant growth [40]. The results of this study showed that growth characteristics and biomass production were significantly reduced and Chl content decreased in tomato leaves subjected to high-temperature stress (Figure S1); exogenous application of Put effectively reduced the heat-stress-induced growth inhibition of tomato plants and simultaneously elevated the Chl content in tomato leaves (Figure 1). These findings indicate that the plant growth promotion function of Put is closely linked to the increase of Chl content in the leaves. Several research works have demonstrated the beneficial roles of exogenous Put application on plant growth attributes, biomass production, and Chl concentration in various crops under different capricious environments [41,42]. Priming with Put had a beneficial impact on leaf FW and DW, which corresponded to an increased salinity tolerance of grapevine [43]. The effects of Put on improving growth parameters can be explained by its polycationic nature and regulation of ion metabolism. 

Maintaining cell membrane function under thermal stress is crucial for photosynthesis and respiration in plants [44]. Heat-induced ROS overproduction impairs photosynthetic organs, causing Chl degradation and hastening the senescence process in plants [45]. Two important enzymes, MgCHT and PBGD, participate in the biosynthesis of Chl and PaO, and Chlase catalyzes chlorophyll degradation [46]. In the current study, the ALA and PBG, along with the activity of PBGD, were significantly reduced in heat-stressed only seedlings; however, the activity of ALA and PBG was markedly elevated in heat-stressed seedlings treated with Put (Figure 2A,B,D). Furthermore, the transcript abundances of *PBGD*, *CHL G*, *CAO*, and *Mg-CHT* were significantly negatively regulated in heat-stressed seedlings (Figure 3A–E). The decrease in Chl biosynthesis in plants is imposed by thermal stress as a result of the reduced activity of enzymes related to Chl biosynthesis [39]. The conversion of Chl b to Chl a during the Chl catabolism process is part of the Chl catabolism pathway [47]. The loss of Chl can stimulate leaf yellowing in higher plants under stress conditions or as part of a natural aging process. This process occurs because Chl metabolic enzymes react with the light harvesting complex of photosystem II (LHCII) and construct protein complexes under stress conditions for degradation of the components of phototoxic intermediates, which leads to Chl degradation [48]. In the current study, Chlase and MDCase activities increased under high-temperature-stressed plants and, correspondingly, the transcript levels of *Chlase* were upregulated under the same stress conditions (Figure 2C,E), which prompts Chl degradation. The rate of Chl degradation was slowed under high-temperature stress, and the accumulation of Chl degradation intermediates was reduced after foliar spraying with Put. It is known that Put has the ability to protect thylakoid membranes via a chlorophyll–protein complex site and has a positive effect on leaf chlorophyll levels [49]. Similarly to our findings, in a high-temperature stress environment, Spd decreases the activity of Chlase and MDCase, as well as the expression of *Chlase*; thus, delaying the enzymatic process of Chl degradation in cucumber [50]. Stay-green reductase (SGR) is linked to the discharge of Chl from the thylakoid membrane. In the present study, heat stress significantly increased *PAO* and *SGR* levels (Figure 3H,I), which can significantly promote Chl loss and possibly increase dissociation of the Chl–protein complex. The addition of Put significantly reduced *SGR* and *PAO* transcription levels under high-temperature stress conditions in tomato plants (Figure 3H,I), indicating that Put suppresses *PAO* expression by downregulating *SGR* expression. It also increases Chl concentration by attenuating the dissociation of Chl protein complexes and restricting Chl degradation. Maintaining optimum Chl content is essential for photosynthesis, when plants are exposed to high-temperature stress. Foliar spraying with Spd could improve the heat tolerance of tomato seedlings that had an optimum photosynthetic capacity [51]. PA-mediated suppression of leaf senescence could extend the photosynthesis process, thus enhancing the starch content of barley [52]. 

Photosynthetic fluorescence is an output of the photosynthetic process that is produced by capturing light energy at the reaction center within the photosynthetic membrane and dissipating it as heat energy after photochemical activity [53]. Heat stress significantly decreased the Fv/Fm, Y(II), and qP values, where NPQ was substantially increased by the same treatment; however, treatment with Put reversed these trends (Figure 5). In a previous study, F0, Fm, Fv/Fm, and ΦPSI were reported to be significantly increased by exogenous Put application, which decreased NPQ and, thus, improved the photosynthetic capacity of plants under stress conditions [28,35,54]. These findings reveal that increased photosynthetic ability is closely linked to lowered oxidative stress, and this increase in response to Put treatment of heat-stressed tomato seedlings resulted from lowered ROS production and cell damage.

Proline, a compatible solute that accumulates under stress conditions, regulates cell membrane permeability and functions in protein stabilization and excess ROS detoxification [55]. In this study, the Put-treated heat-stressed tomato leaves had higher proline levels than those of the control (Figure 6A). In accordance with our findings, some studies have also reported that PAs, including Put, induce proline accumulation and concomitant attenuation of abiotic stress-induced damage in plants through maintenance of membrane integrity, subcellular structure, antioxidant enzymatic activities, and protein structure [38,56]. Under water deficit conditions, similar results were found in wheat, where external Put application had a positive effect on the accumulation of Pro and soluble and insoluble sugars [57]. Previous studies have noted that PAs play a critical role in balancing ROS metabolism under abiotic stress [58]. High-density, positively charged PAs can bind with (negatively charged) phosphate groups on the membrane, resulting in decreased membrane potential and lipid peroxidation levels [59]. PA and Put could substantially reduce ROS in foxtail millet (*Setaria italica* L.) and *Brassica juncea* [60] under salinity stress by increasing antioxidant activities [61]. Alternatively, polyamines have the ability to increase the activities of ROS-scavenging enzymes such as CAT, POD, and SOD. Put supplementation boosted antioxidant enzyme activities and increased osmolyte accumulation, while lowering the MDA and EL levels in our investigation, resulting in more balanced conditions, and reducing heat-induced oxidative damage and improving morphophysiological parameters (Figure 6C,D and Figure 8). Previous studies have also revealed that exogenous Put application enhances antioxidant enzymatic activity and the expression of stress response genes in plants exposed to a variety of abiotic stresses [28].

One of the most important adaptive responses of plants to heat stress is the expression of heat shock proteins (HSPs). When plants are exposed to heat stress, HSPs serve a wide range of functions, including stabilizing denatured proteins, facilitating protein maturation and assembly, reducing protein aggregation, and boosting protein integration and translocation [62,63]. Previous research has demonstrated that extreme temperature, not only increases endogenous PA content, but also upregulates HSP expression in *Arabidopsis* [64]. Under heat stress conditions, cellular PA metabolism is tightly controlled by HSP synthesis, influencing the integrity and attributes of cell membranes in tobacco and alfalfa [63]. PAs have been used to protect cells from heat shock-induced injury by increasing both HSP production and heat-shock-related gene expression in *Arabidopsis* [64]. Exogenous Put could enhance HSP17 transcript abundance, which is associated with improved heat tolerance of wheat seedlings [65]. In the current study, Put application substantially increased the expression of *HSP90* and *HSP70*. This indicates that Put-mediated heat tolerance is involved in the expression of *HSP90* and *HSP70*, which may balance denatured proteins and assist proteins in folding correctly in tomato when exposed to high-temperature stress, suggesting that Put may promote the increased synthesis of Hsps to protect plants from damage (Figure 9). Supplementation with polyamine improves endogenous polyamine content, which can result in a decrease in reactive oxygen species, enhanced plant quality, and even slowing the senescence process [29]. It is worth mentioning that under adverse conditions, Put concentration increases to improve plant tolerance to stressors [38,56]. Foliar spraying of Put mitigates the negative effect of high temperature on cotton flowers and fruit development by enhancing the Put content in cotton flowers, which was associated with a higher seed set [35]. Wheat plants treated with Put before being exposed to heat stress showed increased tolerance to heat stress, most likely due to enhanced endogenous PA and amino acid contents and inhibition of NH_4_^+^ and ethylene production [66]. Previous research has observed that exogenous supplementation with Spd could enhance the PA contents associated with better heat tolerance in tomato seedlings [67]. Under salt stress conditions, exogenous PA application increased the endogenous Put content in rice leaves [68]. Foliar application of Spd significantly increased the contents of free, bound, and conjugated PAs in the leaves of cucumber seedlings under salt-stress conditions [41]. The present results showed that the exogenous addition of Put significantly elevated the endogenous PA content in tomato leaves under heat stress and, thus, increased the heat tolerance of tomato plants (Figure 10). Finally, application of a Put treatment is regarded as a typical strategy to boost the performance of plants exposed to heat stress, primarily by increasing photosynthetic efficiency and antioxidant enzyme activity and restricting chlorophyll loss.

## 4. Materials and Methods

### 4.1. Planting Material and Growth Environments

Tomato (*Solanum lycopersicum* L.) seeds were sanitized with 0.1% sodium hypochlorite (NaOCl) for 5 min, then washed with deionized water several times and kept in the dark at 28 ± 1 °C for 36 h for germination. After germination, the seeds were sown in plastic trays composed of a peat and vermiculite mixture (2:1, *v*:*v*) and cultured in a growth chamber, where growth environments were monitored at 28 ± 1 °C (day) and 19 ± 1 °C (night), relative humidity of 65–75%, and a 12 h photoperiod (PAR 300 µmol m^−2^ s^−1^). After the second true leaf was fully developed, uniformly growing seedlings were transferred to containers filled with a mixture of peat and vermiculite and irrigated with nutrient solution on alternate days. After the fourth true leaf had developed completely, the seedlings were classified into two subgroups for providing Put and high-temperature treatment (38/28 °C temperature (day/night); 14 h/10 h (day/night) photoperiod; 55–65% relative humidity). There were four distinct treatments used in the experiments: (1) control (Cont); (2) putrescine (Put, 1 mM); (3) high temperature (HT, 38/28 °C); and (4) HT (38/28 °C) + Put (1 mM). Every day at 17:00, 1 mM Put was sprayed on leaves, while the other seedlings were sprayed with double distilled water. Following 7 days of treatment, tomato leaves (third from top to bottom) from each treatment were collected and frozen at −80 °C for subsequent analysis.

### 4.2. Analysis of Growth Indicators

Various growth indicators, such as plant height, fresh and dry weight of leaves and roots, and stem diameter were measured to evaluate the combined effect of putrescine and heat stress on tomato seedlings. A standard ruler and a Vernier scale were used for the measurement of plant height and stem diameter, respectively. An electric balance was used to measure the fresh weight of leaves and roots. The plants were oven dried (80 °C for 72 h) before determining the dry weight of leaves and roots.

### 4.3. Determination of Chlorophyll Content and Gas Exchange Parameters

To determine Chl content, leaves were ground in 80% cold acetone and then centrifuged to obtain the supernatant [69], and the Chl content was analyzed using a UV-1800 spectrophotometer. After 7 days of heat treatments, the gas exchange attributes were calculated using a portable infrared gas analysis system (Li-6400; LI-COR, Inc., Lincoln, NE, USA) between 10:00 and 11:00 a.m. [70]. The cuvette specifications for data collection were as follows: 800 μmol photons m^−2^ s^−1^ PPFD (photosynthetic photon flux density), 60–70% relative humidity, 25 °C temperature, and external CO_2_ concentration of 380 ± 10 μmol mol^−1^.

### 4.4. Determination of Chlorophyll Related Enzyme Activity 

Aminolevulinic acid (ALA) concentration was quantified using the protocol described by Klein et al. [71]. Porphobilinogen (PBG) concentration was measured using the method of Bogorad [72]. PBGD was determined according to the method developed by Frydman and Frydman [73]. Chlorophyllase was extracted according to the method prescribed by Fernandez Lopez et al. [74]. The activity of Mg dechelatase (MDCase) was quantified according to the method described by Costa et al. [75].

### 4.5. Measurement of Maximum Photochemical Efficiency

For the measurement of chlorophyll fluorescence, entirely developed leaves were used, and after 30 min dark adaptation, leaf data were collected between 9:00 a.m. to 11:00 a.m. using an IMAGING-PAM Chl fluorescence analyzer (Heinz Walz, Effeltrich, Germany). The Fv/Fm (maximum photochemical efficiency) value was determined in accordance with Maxwell and Johnson [76].

### 4.6. Determination of Proline Accumulation

Proline content was assayed following the method described by Bates et al. [77]. Proline was extracted using 3% sulfosalicylic acid. The extract was diluted in an equal volume of glacial acetic acid and ninhydrin solutions. The sample was placed in a water bath at 100 °C. Subsequently, the sample was kept on an ice and after cool down 5 mL of toluene was added. The reading of the absorbance of the toluene layer was recorded at 520 nm using a spectrophotometer (Spectronic 20D, Milton Roy, Philadelphia, PA, USA). 

### 4.7. Sugar Assays

For the estimation of the total soluble sugar content, dry leaf samples were ground in ethanol and then incubated in a water bath at 80 °C for 30 min. The upper layer was used to determine total soluble sugar [78].

### 4.8. Measurement of Electrolyte Leakage

The electrolyte leakage from foliage was determined according to the method given by Jahan et al. [70]. Briefly, an approximately 10-mm diameter leaf disc was placed on a Petri dish, to block leakage of the electrolyte when the leaf disc was removed, and the leaf was then rinsed with distilled water three times. After that, distilled water was poured into the Petri dish, which was then kept in the dark at room temperature for 24 h. Following incubation, the preliminary electrical conductivity (EC1) of the bath solution was determined. After heating the glass tube to 95 °C in a temperature-controlled water bath for 20 min, the tube was cooled to room temperature and again electrical conductivity (EC2) was measured. Electrolyte leakage was calculated using the following formula:

$$\mathrm{EL}\;\left(\%\right)=\frac{\mathrm{EC}1}{\mathrm{EC}2}\times 100$$

### 4.9. Measurement of Lipid Peroxidation (MDA)

Malondialdehyde (MDA) concentration was quantified using the colorimetric method [79] with minor modifications. In brief, 0.5 g of plant material was macerated in 5 mL of 5% (*w*/*v*) trichloroacetic acid (TCA) solution followed by centrifuging at 4000× *g* at 4 °C for 10 min and with collection of the supernatant. Afterward, 2 mL of TCA containing 0.67% TBA solution mixture was added to the collected supernatant. The mixture was boiled in a water bath (100 °C) for 30 min, before being cooled on ice. The reading of the absorbance of aliquot was recorded at 450, 532, and 600 nm using a spectrophotometer. The MDA content unit was nanomoles per gram of FW.

### 4.10. Histochemical Detection of H_2_O_2_ and O_2_^•−^

The rate of O_2_^•−^ formation and the generation of H_2_O_2_ were measured based on formation of nitro blue tetrazolium (NBT) and 3,3-diamino benzidine (DAB), respectively, according to a previously published method [80] with slight modifications. To localize H_2_O_2_, the stained leaves were implanted in a vacuumed DAB solution and kept for 12 h at room temperature. Brown spots on the surface of the leaves were visible due to the reaction between DAB and H_2_O_2_. For the detection of O_2_^•−^, leaves were immersed in 1 mg·mL^−1^ NBT solution, followed by incubation in a dark place for 12 h under normal conditions. Blue lesions develop in the leaves as a result of the reaction of NBT and O_2_^•−^. To remove excess chlorophyll from the leaves, both stained leaves were washed in a water bath in 95% ethanol for 20 min. Following that, the samples were immersed in absolute ethanol for several hours, prior to being photographed with a digital camera.

### 4.11. H_2_O_2_ Measurement

H_2_O_2_ concentration in leaves was measured based on the method developed by Ma et al. [81]. Briefly, the plants were ground in 2 mL of cold acetone, then centrifuged at 10,000× *g* for 15 min, followed by collection of the supernatant. The supernatant was then mixed with 0.05 mL of TiCl4 (20%) solution and 0.1 mL of concentrated ammonia water, then centrifuged at 3000× *g* for 10 min. Then, the remaining residual was washed three times with cold acetone, and 3 mL of 2 M H_2_SO_4_ was added to dissolve the precipitate. The absorbance values were measured at 415 nm using a spectrophotometer. 

### 4.12. Determination of O_2_^•−^ Production Rate

The rate of O_2_^•−^ production was determined according to He et al. [82], with some alterations. In brief, 0.2 g of leaves tissues were macerated in 2 mL of 50 mM phosphate buffer (pH 7.8) and centrifuged at 12,000× *g* for 20 min at 4 °C. After that, 0.5 mL of 50 mM phosphate buffer (pH 7.8) and 0.1 mL of 10 mM hydroxylamine hydrochloride were added to 0.5 mL of supernatant followed by incubation at room temperature for 30 min. After incubation, 1 mL of 17 mM sulfanilamide and 1 mL of 7 mM naphthylamine were incorporated into the mixture solution and further incubated for 30 min. The absorbance reading was taken at 530 nm. 

### 4.13. Antioxidant Enzyme Assay

To determine the antioxidant enzymes activity, 0.2 g of composite leaf tissues was homogenized in precooled 1.6 mL of 50 mM phosphate buffer (pH 7.8) and centrifuged at 12,000× *g* for 20 min at 4 °C, followed by collecting the supernatant. The superoxide dismutase (SOD, EC 1.15.1.1) activity was determined according to the method of Giannopolitis and Ries [83]. The activity of peroxidase (POD, EC 1.11.1.7) was determined based on the method described by Tao et al. [84]. The method of Dindsa et al. [85] was used to determine the catalase activity (CAT, EC 1.11.1.6). To determine the activity of ascorbic acid oxidase (APX), the method described by Nakano et al. [86] was used. 

Monodehydroascorbate reductase activity (MDHAR, EC 1.6.5.4) was determined as previously described by Hossain et al. [87]. Dehydroascorbate reductase activity (DHAR, EC 1.8.5.1) was determined in accordance with the method developed by Nakano et al. [86]. 

The activity of glutathione reductase (GR) was determined using a GR detection kit (Solarbio Life Science, Beijing, China), according to the manufacturer’s protocols. Glutathione S-transferase (GST) activity was measured using a GST assay kit (Solarbio Life Science, Beijing, China). Lipoxygenase (LOX) activity was quantified using a lipoxygenase detection kit (Solarbio Life Science, Beijing, China), according to the company’s instructions.

### 4.14. Protein Extraction 

The protein content was estimated according to the method described by Bradford [88].

### 4.15. Determination of Free Polyamines Content

Polyamine contents were determined according to the procedure described by Shen et al. [34], with a few modifications. In summary, 0.5 g of composite leaf tissue was ground in 5% (*v*/*v*) precooled perchloric acid followed by incubation on ice for 1 h. Subsequently, homogenates were centrifuged at 12,000× *g* for 20 min, and the upper layer was used to estimate free PAs. A 0.7 mL aliquot of supernatant was mixed with 1.4 mL of NaOH (2N) and 15 μL of benzoyl chloride, and then gently shaken in a vortex mixer, followed by incubated at 37 °C for 30 min. Saturated NaCl (2 mL) was added to the reaction mixture to stop the reaction. For the extraction of benzoyl-PA, 2 mL of cold diethyl ether was added followed by centrifuging at 3000× *g* for 5 min. After extraction, the benzoyl-PAs were redissolved in 1 mL of 64% (*v*/*v*) methanol. To isolate and determine the PA content, UHPLC (ultimate high performance liquid chromatograph, Ultimate 3000, Thermo Scientific, San Jose, CA, USA) was applied with a C18 column at a flow rate of 0.8 mL·min^−1^.

### 4.16. Total RNA Isolation and Gene Expression Analysis

Around 100 mg of composite tomato leaves were used to extract total RNA, using an RNAsimple Total RNA Kit (TIANGEN, Beijing, China) according to the manufacturer’s instructions. As per the manufacturer guidelines, 1 µg of total RNA was reverse-transcribed into cDNA for quantitative real-time PCR using a SuperScript First-strand Synthesis System (Takara, Tokyo, Japan). DNA sequences were used to design gene-specific primers, and primer pair sequences are listed in Table S1. A StepOnePlus™ Real-Time PCR system (Applied Biosystems, Foster City, CA, USA) was employed to carry out Real-time PCR with ChamQ Universal SYBR qPCR Master Mix (Vazyme Biotech Co., Ltd., Nanjing, China). The relative gene expression was computed according to Liu et al. [89], and mRNA expression levels were normalized against actin and then compared.

### 4.17. Statistical Analysis

At least three independent biological replicates were studied for all treatments and measurements. All data were statistically analyzed using the Statistical Package for the Social Sciences (SPSS 20.0 version, SPSS Inc., Chicago, IL, USA), and the results are presented as means ± SDs. One-way analysis of variance (ANOVA) was used to analyze data, and the mean differences among the treatments were calculated using Tukey’s honesty significant test (HSD) at *p* < 0.05. The graphs were generated using Origin Pro 9.0.

## 5. Conclusions

Foliar application of Put can significantly enhance the heat tolerance of tomato. Put treatments elevated free Put, Spd content, and the activity of Chl enzymes, and decreased Spm levels, under thermal stress. Put supplementation increased antioxidant enzyme activity and decreased the levels of oxidative stress markers (MDA, H_2_O_2_, and O_2_^•−^); thus, efficiently mitigating temperature-induced oxidative damage. Furthermore, Put treatment of tomato plants resulted in maintenance of a significantly higher level of photosynthesis and photosynthetic pigment content than in heat-stressed only plants, which could be attributed to the functions of Put in elevating Chl synthesis and restricting Chl deterioration by activating Chl enzyme activity and suppressing the expression of Chl catabolism genes. In addition, Put-induced heat tolerance is implicated in promoting the expression of *HSP90* and *HSP70*, which may function by balancing protein degradation and assisting proteins to fold correctly in tomato seedlings exposed to heat stress conditions and, working together, enhance the heat tolerance of tomato seedlings.

## Data Availability

The data presented in this study are available on request from the corresponding author.

## Supplementary Materials

The following supporting information can be downloaded at https://www.mdpi.com/article/10.3390/plants11081038/s1, Figure S1: Interactive effects of Put and high temperature on growth parameters in tomato seedlings. Table S1: List of primers used in this study.

## References

[1] Jha U.C., Bohra A., Singh N.P. (2014). Heat stress in crop plants: Its nature, impacts and integrated breeding strategies to improve heat tolerance. Plant Breed..

[2] Parry I.W., Pizer W.A. (2007). Combating global warming. Regulatio.

[3] Battisti D.S., Naylor R.L. (2009). Historical warnings of future food insecurity with unprecedented seasonal heat. Science.

[4] Jahan M.S., Shu S., Wang Y., Chen Z., He M., Tao M., Sun J., Guo S. (2019). Melatonin alleviates heat-induced damage of tomato seedlings by balancing redox homeostasis and modulating polyamine and nitric oxide biosynthesis. BMC Plant Biol..

[5] Vollenweider P., Günthardt-Goerg M.S. (2005). Diagnosis of abiotic and biotic stress factors using the visible symptoms in foliage. Environ. Pollut..

[6] Wahid A., Gelani S., Ashraf M., Foolad M.R. (2007). Heat tolerance in plants: An overview. Environ. Exp. Bot..

[7] Muhammad I., Yang L., Ahmad S., Mosaad I.S.M., Al-Ghamdi A.A., Abbasi A.M., Zhou X.B. (2022). Melatonin application alleviates stress-induced photosynthetic inhibition and oxidative damage by regulating antioxidant defense system of maize: A meta-analysis. Antioxidants.

[8] Gill S.S., Tuteja N. (2010). Reactive oxygen species and antioxidant machinery in abiotic stress tolerance in crop plants. Plant Physiol. Biochem..

[9] Muhammad I., Yang L., Ahmad S., Farooq S., Al-Ghamdi A.A., Khan A., Zeeshan M., Elshikh M.S., Abbasi A.M., Zhou X.B. (2022). Nitrogen fertilizer modulates plant growth, chlorophyll pigments and enzymatic activities under different irrigation regimes. Agronomy.

[10] Kumar Tewari A., Charan Tripathy B. (1998). Temperature-stress-induced impairment of chlorophyll biosynthetic reactions in cucumber and wheat. Plant Physiol..

[11] Nagata N., Tanaka R., Satoh S., Tanaka A. (2005). Identification of a vinyl reductase gene for chlorophyll synthesis in Arabidopsis thaliana and implications for the evolution of Prochlorococcus species. Plant Cell.

[12] Matile P., Hörtensteiner S., Thomas H. (1999). Chlorophyll degradation. Annu. Rev. Plant Biol..

[13] Naor V., Kigel J. (2002). Temperature affects plant development, flowering and tuber dormancy in calla lily (*Zantedeschia*). J. Hortic. Sci. Biotec..

[14] Camejo D., Jiménez A., Alarcón J.J., Torres W., Gómez J.M., Sevilla F. (2006). Changes in photosynthetic parameters and antioxidant activities following heat-shock treatment in tomato plants. Funt. Plant Biol..

[15] Sharkey T.D., Zhang R. (2010). High temperature effects on electron and proton circuits of photosynthesis. J. Integr. Plant Biol..

[16] Ruan Y.L., Jin Y., Yang Y.J., Li G.J., Boyer J.S. (2010). Sugar input, metabolism, and signaling mediated by invertase: Roles in development, yield potential, and response to drought and heat. Mol. Plant.

[17] Scharf K.D., Berberich T., Ebersberger I., Nover L. (2012). The plant heat stress transcription factor (Hsf) family: Structure, function and evolution. Biochim. Biophys. Acta Gene Regul. Mech. BBA-Gene Regul. Mech..

[18] Ohama N., Sato H., Shinozaki K., Yamaguchi-Shinozaki K. (2017). Transcriptional regulatory network of plant heat stress response. Trends Plant Sci..

[19] Wu J., Shu S., Li C., Sun J., Guo S. (2018). Spermidine-mediated hydrogen peroxide signaling enhances the antioxidant capacity of salt-stressed cucumber roots. Plant Physiol. Biochem..

[20] Liu J.H., Wang W., Wu H., Gong X., Moriguchi T. (2015). Polyamines function in stress tolerance: From synthesis to regulation. Front. Plant Sci..

[21] Fu X.Z., Xing F., Wang N.Q., Peng L.Z., Chun C.P., Cao L., Ling L.L., Jiang C.L. (2014). Exogenous spermine pretreatment confers tolerance to combined high-temperature and drought stress in vitro in trifoliate orange seedlings via modulation of antioxidative capacity and expression of stress-related genes. Biotechnol. Biotechnol. Equip..

[22] Yamakawa H., Kamada H., Satoh M., Ohashi Y. (1998). Spermine is a salicylate-independent endogenous inducer for both tobacco acidic pathogenesis-related proteins and resistance against tobacco mosaic virus infection. Plant Physiol..

[23] Hussain S.S., Ali M., Ahmad M., Siddique K.H. (2011). Polyamines: Natural and engineered abiotic and biotic stress tolerance in plants. Biotechnol. Adv..

[24] Pang X.M., Zhang Z.Y., Wen X.-P., Ban Y., Moriguchi T. (2007). Polyamines, all-purpose players in response to environment stresses in plants. Plant Stress.

[25] Mostafaei E., Zehtab-Salmasi S., Salehi-Lisar Y., Ghassemi-Golezani K. (2018). Changes in photosynthetic pigments, osmolytes and antioxidants of Indian Mustard by drought and exogenous polyamines. Acta Biol. Hung..

[26] Collado-González J., Piñero M.C., Otálora G., López-Marín J., del Amor F.M. (2021). Effects of Different Nitrogen Forms and Exogenous Application of Putrescine on Heat Stress of Cauliflower: Photosynthetic Gas Exchange, Mineral Concentration and Lipid Peroxidation. Plants.

[27] Li X., Gong B., Xu K. (2015). Effects of exogenous spermidine on endogenous hormone and chloroplast ultrastructure of ginger leaves under high temperature stress. Sci. Agric. Sin..

[28] Jing J., Guo S., Li Y., Li W. (2019). Effects of polyamines on agronomic traits and photosynthetic physiology of wheat under high temperature stress. Photosynthetica.

[29] Chen D., Shao Q., Yin L., Younis A., Zheng B. (2019). Polyamine function in plants: Metabolism, regulation on development, and roles in abiotic stress responses. Front. Plant Sci..

[30] Liu K., Fu H., Bei Q., Luan S. (2000). Inward potassium channel in guard cells as a target for polyamine regulation of stomatal movements. Plant Physiol..

[31] Islam M., Ryu B.R., Azad M., Kalam O., Rahman M., Rana M., Lim J.-D., Lim Y.-S. (2021). Exogenous putrescine enhances salt tolerance and ginsenosides content in Korean ginseng (*Panax ginseng* Meyer) sprouts. Plants.

[32] Yuan Y., Shu S., Li S., He L., Li H., Du N., Sun J., Guo S. (2014). Effects of exogenous putrescine on chlorophyll fluorescence imaging and heat dissipation capacity in cucumber (*Cucumis sativus* L.) under salt stress. J. Plant Growth Regul..

[33] Zhang R.H., Li J., Guo S.R., Tezuka T. (2009). Effects of exogenous putrescine on gas-exchange characteristics and chlorophyll fluorescence of NaCl-stressed cucumber seedlings. Photosynth. Res..

[34] Shen J.l., Wang Y., Shu S., Jahan M.S., Zhong M., Wu J.Q., Sun J., Guo S.R. (2019). Exogenous putrescine regulates leaf starch overaccumulation in cucumber under salt stress. Sci. Hortic..

[35] Bibi A., Oosterhuis D., Gonias E. (2010). Exogenous application of putrescine ameliorates the effect of high temperature in Gossypium hirsutum L. flowers and fruit development. J. Agron. Crop Sci..

[36] Shu S., Yuan Y., Chen J., Sun J., Zhang W., Tang Y., Zhong M., Guo S. (2015). The role of putrescine in the regulation of proteins and fatty acids of thylakoid membranes under salt stress. Sci. Rep..

[37] Cui J., Pottosin I., Lamade E., Tcherkez G. (2020). What is the role of putrescine accumulated under potassium deficiency?. Plant Cell Environ..

[38] Islam M.J., Uddin M.J., Hossain M.A., Henry R., Begum M.K., Sohel M.A.T., Mou M.A., Ahn J., Cheong E.J., Lim Y.-S. (2022). Exogenous putrescine attenuates the negative impact of drought stress by modulating physio-biochemical traits and gene expression in sugar beet (*Beta vulgaris* L.). PLoS ONE.

[39] Reda F., Mandoura H.M. (2011). Response of enzymes activities, photosynthetic pigments, proline to low or high temperature stressed wheat plant (*Triticum aestivum* L.) in the presence or absence of exogenous proline or cysteine. Int. J. Acad. Res..

[40] Seemann J.R., Berry J.A., Downton W.J.S. (1984). Photosynthetic response and adaptation to high temperature in desert plants: A comparison of gas exchange and fluorescence methods for studies of thermal tolerance. Plant Physiol..

[41] Shu S., Guo S.R., Sun J., Yuan L.Y. (2012). Effects of salt stress on the structure and function of the photosynthetic apparatus in Cucumis sativus and its protection by exogenous putrescine. Physiol. Plant..

[42] Demetriou G., Neonaki C., Navakoudis E., Kotzabasis K. (2007). Salt stress impact on the molecular structure and function of the photosynthetic apparatus—the protective role of polyamines. Biochim. Biophys. Acta-Bioenerg..

[43] Gohari G., Panahirad S., Sadeghi M., Akbari A., Zareei E., Zahedi S.M., Bahrami M.K., Fotopoulos V. (2021). Putrescine-functionalized carbon quantum dot (put-CQD) nanoparticles effectively prime grapevine (*Vitis vinifera* cv.‘Sultana’) against salt stress. BMC Plant Biol..

[44] Chen J., Wang P., Mi H.l., Chen G.Y., Xu D.Q. (2010). Reversible association of ribulose-1, 5-bisphosphate carboxylase/oxygenase activase with the thylakoid membrane depends upon the ATP level and pH in rice without heat stress. J. Exp. Bot..

[45] Jespersen D., Huang B. (2015). Proteins associated with heat-induced leaf senescence in creeping bentgrass as affected by foliar application of nitrogen, cytokinins, and an ethylene inhibitor. Proteomics.

[46] Hu L., Xiang L., Li S., Zou Z., Hu X.H. (2016). Beneficial role of spermidine in chlorophyll metabolism and D1 protein content in tomato seedlings under salinity–alkalinity stress. Physiol. Plant..

[47] Vezitskii A.Y., Shcherbakov R. (2002). Interconversions of chlorophylls a and b synthesized from exogenous chlorophyllides a and b in etiolated and post-etiolated rye seedlings. Russ. J. Plant Physiol..

[48] Sakuraba Y., Kim D., Kim Y.S., Hörtensteiner S., Paek N.C. (2014). Arabidopsis STAYGREEN-LIKE (SGRL) promotes abiotic stress-induced leaf yellowing during vegetative growth. FEBS Lett..

[49] Besford R., Richardson C., Campos J., Tiburcio A. (1993). Effect of polyamines on stabilization of molecular complexes in thylakoid membranes of osmotically stressed oat leaves. Planta.

[50] Zhou H., Guo S., An Y., Shan X., Wang Y., Shu S., Sun J. (2016). Exogenous spermidine delays chlorophyll metabolism in cucumber leaves (*Cucumis sativus* L.) under high temperature stress. Acta Physiol. Plant..

[51] Murkowski A. (2001). Heat stress and spermidine: Effect on chlorophyll fluorescence in tomato plants. Biol. Plant..

[52] Sobieszczuk-Nowicka E., Paluch-Lubawa E., Mattoo A.K., Arasimowicz-Jelonek M., Gregersen P.L., Pacak A. (2019). Polyamines–A new metabolic switch: Crosstalk with networks involving senescence, crop improvement, and mammalian cancer therapy. Front. Plant Sci..

[53] Thwe A.A., Kasemsap P. (2014). Quantification of OJIP fluorescence transient in tomato plants under acute ozone stress. Agric. Nat. Res..

[54] Khoshbakht D., Asghari M., Haghighi M. (2018). Influence of foliar application of polyamines on growth, gas-exchange characteristics, and chlorophyll fluorescence in Bakraii citrus under saline conditions. Photosynthetica.

[55] Hayat S., Hayat Q., Alyemeni M.N., Wani A.S., Pichtel J., Ahmad A. (2012). Role of proline under changing environments: A review. Plant Signal. Behav..

[56] Tanou G., Ziogas V., Belghazi M., Christou A., Filippou P., Job D., Fotopoulos V., Molassiotis A. (2014). Polyamines reprogram oxidative and nitrosative status and the proteome of citrus plants exposed to salinity stress. Plant Cell Environ..

[57] Abd Elbar O.H., Farag R.E., Shehata S.A. (2019). Effect of putrescine application on some growth, biochemical and anatomical characteristics of *Thymus vulgaris* L. under drought stress. Ann. Agric. Sci..

[58] Andronis E.A., Moschou P.N., Toumi I., Roubelakis-Angelakis K.A. (2014). Peroxisomal polyamine oxidase and NADPH-oxidase cross-talk for ROS homeostasis which affects respiration rate in *Arabidopsis thaliana*. Front. Plant Sci..

[59] Mathaba N., Tesfay S., Bower J., Bertling I. Effect of hot water and molybdenum dips on endogenous polyamines and heat shock protein (HSP70) in lemon flavedo and their ability to alleviate chilling injury during cold storage. Proceedings of the VII International Postharvest Symposium 1012.

[60] Sudhakar C., Veeranagamallaiah G., Nareshkumar A., Sudhakarbabu O., Sivakumar M., Pandurangaiah M., Kiranmai K., Lokesh U. (2015). Polyamine metabolism influences antioxidant defense mechanism in foxtail millet (*Setaria italica* L.) cultivars with different salinity tolerance. Plant Cell Rep..

[61] Verma S., Mishra S.N. (2005). Putrescine alleviation of growth in salt stressed *Brassica juncea* by inducing antioxidative defense system. J. Plant Physiol..

[62] Jahan M.S., Guo S., Sun J., Shu S., Wang Y., El-Yazied A.A., Alabdallah N.M., Hikal M., Mohamed M.H.M., Ibrahim M.F.M. (2021). Melatonin mediated photosynthetic performance of tomato seedlings under high temperature stress. Plant Physiol. Biochem..

[63] Wang C., Fan L., Gao H., Wu X., Li J., Lv G., Gong B. (2014). Polyamine biosynthesis and degradation are modulated by exogenous gamma-aminobutyric acid in root-zone hypoxia-stressed melon roots. Plant Physiol. Biochem..

[64] Sagor G., Berberich T., Takahashi Y., Niitsu M., Kusano T. (2013). The polyamine spermine protects Arabidopsis from heat stress-induced damage by increasing expression of heat shock-related genes. Transgenic Res..

[65] Kumar R.R., Singh G., Sharma S.K., Singh K., Goswami S., Rai R.D. (2012). Molecular cloning of HSP17 gene (sHSP) and their differential expression under exogenous putrescine and heat shock in wheat (*Triticum aestivum*). Afr. J. Biotechnol..

[66] Hassanein R.A., El-Khawas S.A., Ibrahim S.K., El-Bassiouny H.M., Mostafa H., Abdel-Monem A.A. (2013). Improving the thermo tolerance of wheat plant by foliar application of arginine or putrescine. Pak. J. Bot..

[67] Sang Q., Shu S., Shan X., Guo S., Sun J. (2016). Effects of exogenous spermidine on antioxidant system of tomato seedlings exposed to high temperature stress. Russ. J. Plant Physiol..

[68] Roychoudhury A., Basu S., Sengupta D.N. (2011). Amelioration of salinity stress by exogenously applied spermidine or spermine in three varieties of indica rice differing in their level of salt tolerance. J. Plant Physiol..

[69] Jabborova D., Ma H., Bellingrath-Kimura S.D., Wirth S. (2021). Impacts of biochar on basil (*Ocimum basilicum*) growth, root morphological traits, plant biochemical and physiological properties and soil enzymatic activities. Sci. Hortic..

[70] Jahan M.S., Wang Y., Shu S., Zhong M., Chen Z., Wu J., Sun J., Guo S. (2019). Exogenous salicylic acid increases the heat tolerance in Tomato (*Solanum lycopersicum* L.) by enhancing photosynthesis efficiency and improving antioxidant defense system through scavenging of reactive oxygen species. Sci. Hortic..

[71] Klein S., Harel E., Ne’Eman E., Katz E., Meller E. (1975). Accumulation of δ-aminolevulinic acid and its relation to chlorophyll synthesis and development of plastid structure in greening leaves. Plant Physiol..

[72] Bogorad L. (1962). Porphyrin synthesis. Methods in Enzymology.

[73] Frydman R.B., Frydman B. (1979). Disappearance of porphobilinogen deaminase activity in leaves before the onset of senescence. Plant Physiol..

[74] Fernandez-Lopez J., Almela L., Lopez-Roca J., Alcaraz C. (1991). Iron deficiency in citrus limon: Effects on photo-chlorophyllase synthetic pigments and chlorophyllase activity. J. Plant Nutr..

[75] Costa M.L., Civello P.M., Chaves A.R., Martínez G.A. (2002). Characterization of Mg-dechelatase activity obtained from Fragaria× ananassa fruit. Plant Physiol. Biochem..

[76] Maxwell K., Johnson G.N. (2000). Chlorophyll fluorescence—A practical guide. J. Exp. Bot..

[77] Bates L.S., Waldren R.P., Teare I. (1973). Rapid determination of free proline for water-stress studies. Plant Soil.

[78] Yang L., Chi Y.X., Wang Y.F., Zeeshan M., Zhou X.B. (2021). Gradual application of potassium fertilizer elevated the sugar conversion mechanism and yield of waxy and sweet fresh-eaten maize in the semiarid cold region. J. Food Quality.

[79] Heath R.L., Packer L. (1968). Photoperoxidation in isolated chloroplasts: I. Kinetics and stoichiometry of fatty acid peroxidation. Arch. Biochem. Biophys..

[80] Ahmad S., Wang G.Y., Muhammad I., Chi Y.X., Zeeshan M., Nasar J., Zhou X.B. (2022). Interactive effects of melatonin and nitrogen improve drought tolerance of maize seedlings by regulating growth and physiochemical attributes. Antioxidants.

[81] Ma S., Zhou X., Jahan M.S., Guo S., Tian M., Zhou R., Liu H., Feng B., Shu S. (2022). Putrescine regulates stomatal opening of cucumber leaves under salt stress via the H_2_O_2_-mediated signaling pathway. Plant Physiol. Biochem..

[82] He M., Jahan M.S., Wang Y., Sun J., Shu S., Guo S. (2020). Compost amendments based on vinegar residue promote tomato growth and suppress bacterial wilt caused by *Ralstonia Solanacearum*. Pathogens.

[83] Giannopolitis C.N., Ries S.K. (1977). Superoxide dismutases: I. Occurrence in higher plants. Plant Physiol..

[84] Tao M.Q., Jahan M.S., Hou K., Shu S., Wang Y., Sun J., Guo S.R. (2020). Bitter melon (*Momordica charantia* L.) rootstock improves the heat tolerance of cucumber by regulating photosynthetic and antioxidant defense pathways. Plants.

[85] Dhindsa R.S., Plumb-Dhindsa P.L., Reid D.M. (1982). Leaf senescence and lipid peroxidation: Effects of some phytohormones, and scavengers of free radicals and singlet oxygen. Physiol. Plant..

[86] Nakano Y., Asada K. (1981). Hydrogen peroxide is scavenged by ascorbate-specific peroxidase in spinach chloroplasts. Plant Cell Physiol..

[87] Hossain M.A., Nakano Y., Asada K. (1984). Monodehydroascorbate reductase in spinach chloroplasts and its participation in regeneration of ascorbate for scavenging hydrogen peroxide. Plant Cell Physiol..

[88] Bradford M.M. (1976). A rapid and sensitive method for the quantitation of microgram quantities of protein utilizing the principle of protein-dye binding. Anal. Biochem..

[89] Liu X., Chen Z., Jahan M.S., Wen Y., Yao X., Ding H., Guo S., Xu Z. (2020). RNA-Seq analysis reveals the growth and photosynthetic responses of rapeseed (*Brassica napus* L.) under red and blue LEDs with supplemental yellow, green, or white light. Hortic. Res..

