# Supplementary material for: Exogenous Putrescine Increases Heat Tolerance in Tomato Seedlings by Regulating Chlorophyll Metabolism and Enhancing Antioxidant Defense Efficiency
Source: Plants (Basel). 2022 Apr 11;11(8):1038. doi: 10.3390/plants11081038 (PMC9032913; doi:10.3390/plants11081038)
Supplement: Supplementary file 1 [file plants-11-01038-s001.zip › plants-1611680-supplementary.pdf]

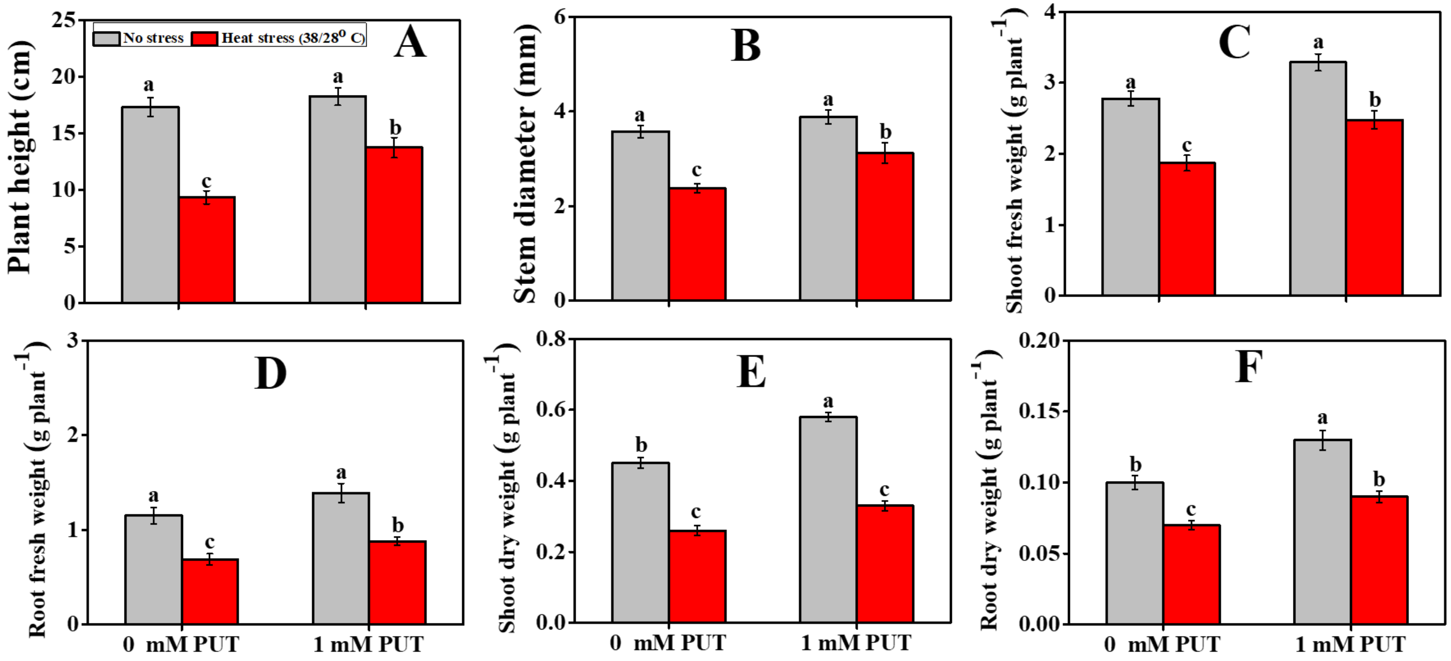

Figure S1. Interactive effects of Put and high temperature on growth parameters in tomato seedlings. (**A**) Plant height, (**B**) Stem diameter, (**C**) Shoot fresh weight, (**D**) Root fresh weight, (**E**) Shoot dry weight and (**F**) Root dry weight. The data denote the mean value ± standard error (n = 3). Different alphabetic letters represent the significant differences among the treatments at $P < 0.05$, according to Tukey's test.

Table S1. List of primers used in this study.

Gene Acronym	Accession Number	Forward Primer	Reverse Primer
FeSOD	Solyc06g048410	GCAGTCAGTATTAGGCTTA	CCATCAACTACATAAACAACA
MnSOD	XM_004240820	GGAACACGCATACTACTT	GTAAACATCATTGGCATATTTC
CAT	Solyc12g094620	GCAGCTCCCAGTTAATGCTC	AGCAGGACGACAAGGATCAA
POD	Solyc07g052510	GGTCTGTTCCAATCCGATGC	CACCAGCACTCCCTGTCTTA
APX	Solyc06g005150	GGCACTCTGCTGGTACCTAT	GGAGAGAGTGGGAAACTGCT
GR	Solyc09g065900	GGAGCCATAGAGGTTGACGA	CTCCTCCCTCCATCAAAGCA
MDHAR	Solyc08g081530	CGGACAGTTCCGAACAAACA	CCCGTGCAATTCGGTTGTAT
DHAR	Solyc05g054760	GAGGTGAACCCTGAAGGGAA	CCCACAGAGGCAAATTCAGG
LOX	Solyc01g099190	TTGGCTTATACTCTTACG	GAATACCTTGTCTGGATT
HSP90	Solyc05g010670	GCAATGGCTCTTGGAGGAAG	CGGTCCTTACTCAGATGGCT
HSP70	Solyc04g009320	AGATGTATAATGCGATGGAT	ATTGCTTCACTCACTCAT
HSfA1	Solyc08g005170	CCCAGTGCTACAGGAAGTGA	TCATCAGCCACTGGTTCCAT
SGR	Solyc06g069640	TTCTCAGTTGCAAGGTTGGT	CCTTGAGAACCACAGGGAGT
PAO	Solyc04g039880	AAATGCCCTCATCGTCTTGC	AGCACAAGCTCTTGGAGACT
NYC1	Solyc07g024000	CAGGCAGCTTCAATCATCCC	TAGGTCGGTTAGGACCATGC
POR	Solyc12g013710	ATGATTGACGGTGGTGACTTTG	CTCCCTGAATAGCCCTGTTGTC
CAO	Solyc04g024920	CACGAAGAACGCTGAATGTCTC	GCTCAAAGCAATCAATCGGTAAC
CHL G	Solyc09g014760	GGTCTGGGCATAGCCATTGT	CTGGAAGTGACTGAAGCCCC
PBGD	Solyc07g066470	TGGAGCAGATGTTGTCAGTC	GCATACTTCGCTGTGTAAGAC
MG-CHT	XM_015217369.1	AAGCACCTGGTAATCTGAACTCTG	CATCGGGTCACCTTCGTATC
CHLASE	Solyc06g053980	CATAGCCGTGGGGGAAAAGT	TGAAATCATTGGAGGAGTCTTTGC
ACTIN	Solyc03g078400	TGGTCGGAATGGGACAGAAG	CTCAGTCAGGAGAACAGGGT
